# A calmodulin like EF hand protein positively regulates oxalate decarboxylase expression by interacting with E-box elements of the promoter

**DOI:** 10.1038/srep14578

**Published:** 2015-10-12

**Authors:** Ayushi Kamthan, Mohan Kamthan, Avinash Kumar, Pratima Sharma, Sekhu Ansari, Sarjeet Singh Thakur, Abira Chaudhuri, Asis Datta

**Affiliations:** 1 National Institute of Plant Genome Research, New Delhi, India

## Abstract

Oxalate decarboxylase (*OXDC*) enzyme has immense biotechnological applications due to its ability to decompose anti-nutrient oxalic acid. *Flammulina velutipes*, an edible wood rotting fungus responds to oxalic acid by induction of *OXDC* to maintain steady levels of pH and oxalate anions outside the fungal hyphae. Here, we report that upon oxalic acid induction, a calmodulin (CaM) like protein-Fv*CaMLP*, interacts with the *OXDC* promoter to regulate its expression. Electrophoretic mobility shift assay showed that FvCamlp specifically binds to two non-canonical E-box elements (AACGTG) in the *OXDC* promoter. Moreover, substitutions of amino acids in the EF hand motifs resulted in loss of DNA binding ability of FvCamlp. *F. velutipes* mycelia treated with synthetic siRNAs designed against Fv*CaMLP* showed significant reduction in Fv*CaMLP* as well as *OXDC* transcript pointing towards positive nature of the regulation. Fv*CaMLP* is different from other known EF hand proteins. It shows sequence similarity to both CaMs and myosin regulatory light chain (Cdc4), but has properties typical of a calmodulin, like binding of ^45^Ca^2+^, heat stability and Ca^2+^ dependent electrophoretic shift. Hence, FvCaMLP can be considered a new addition to the category of unconventional Ca^2+^ binding transcriptional regulators.

The effects of changes in intracellular Ca^2+^ levels are mediated primarily by Ca^2+^-binding proteins that belong to the EF hand super family^1^. EF hand proteins have been classified into 45 distinct subfamilies, 13 of which are congruent with each other and are known as CTER-calmodulin, troponin C, essential light chain, and regulatory light chain subfamilies^2^,^3^,^4^. Calmodulin is one of the most important mediators of calcium signals in the eukaryotic cells^5^,^6^,^7^. CaM is one of the most studied Ca^2+^- sensing protein that is strikingly conserved throughout the evolution at both structural and sequence level^1^. Cdc4 is another class of protein belonging to EF hand super family. It bears significant sequence similarity to both regulatory and essential myosin light chains from diverse eukaryotes. In fission yeast, myosin light chain Cdc4 has been observed to be localized in the medial actin contractile ring playing an essential role in cytokinesis^8^. Interestingly, studies have shown that some proteins with EF hands have the ability to directly interact with DNA. In addition to several Ca^2+^ binding transcription factors^9^,^10^, calmodulin (CaM), a ubiquitous Ca^2+^-binding protein is known to affect gene expression by indirect modulation of other proteins or by direct binding to promoter. One of the CaM isoforms (*CAM7*) of *Arabidopsis thaliana* has been shown to function as a transcriptional regulator by directly binding to a Z-/G-box element located in the promoter of light-responsive genes, and triggering their expression^11^.

Oxalate decarboxylase (*OXDC*) is an oxalate degrading enzyme with potential use in the diagnostics^12^, therapeutics^13^, process industry^14^, and agriculture^15^. *OXDC* from the wood-rotting fungus *F. velutipes* was shown to be induced at the transcriptional level by oxalic acid^16^ to maintain steady extracellular pH. Moreover, in a later study, it was shown that induction of *OXDC* is pH dependent and can occur even in presence of acid like HCl^17^. It was demonstrated that acid-induced cells of *F. velutipes* contain a LPRF (low pH responsive factor) that specifically binds to a 13 bp sequence (5′-GCGGGGTCGCCGA-3′) in 5′ upstream region of *OXDC* gene.

Here, we show that in presence of oxalic acid, a calmodulin like EF hand protein from *F. velutipes-* Fv*CaMLP*, directly interacts with the promoter of *OXDC* and enhances its expression. Though, Fv*CaMLP* shows sequence similarity to both calmodulins (CaMs) and myosin regulatory light chain (Cdc4), but has properties typical of a CaM. Fv*CaMLP* can be considered a new addition to the category of unconventional Ca^2+^ binding transcriptional regulators. Fv*CaMLP* was isolated in yeast one hybrid screening using 100 bp region of *OXDC* promoter as bait, performed with an aim to isolate the proteins regulating the *OXDC* transcription. Electrophoretic mobility shift assay (EMSA) showed that FvCamlp specifically binds to two non-canonical E-box elements (AACGTG) in the *OXDC* promoter and amino acid residues from EF hand motifs of the protein are involved in DNA binding. *F. velutipes* mycelia treated with synthetic siRNAs designed against Fv*CaMLP* showed significant reduction in Fv*CaMLP* as well as *OXDC* transcript, thus confirming its positive nature of regulation.

## Results

### Effect of oxalic acid on mycelial growth and *OXDC* expression

**Figure 1:**
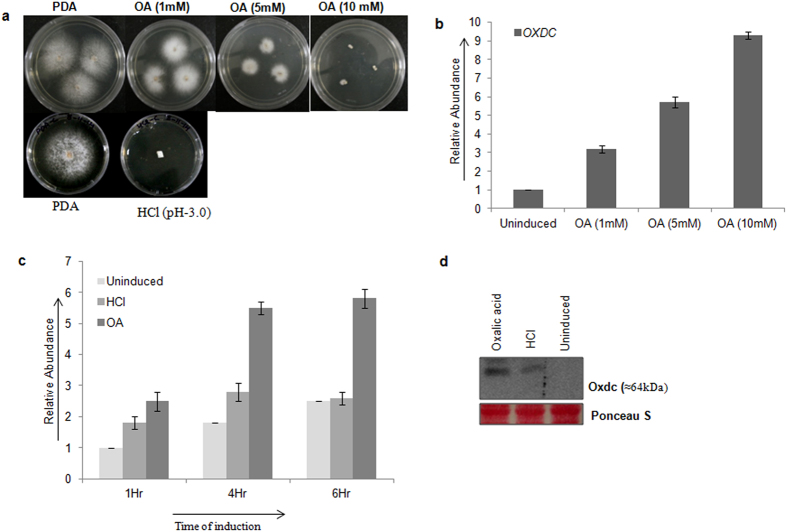
Effect of oxalic acid on *F. velutipes* growth and *OXDC* expression. (**a**) Morphology of *F. velutipes* on PDA (potato dextrose agar) medium consisting of different concentrations of oxalic acid (1 mM, 5 mM & 10 mM) and HCl (pH. 3.0). (**b**) qRT-PCR analysis to compare the expression of *OXDC* at indicated concentration of oxalic acid. (**c**) qRT-PCR analysis to compare *OXDC* expression in presence of HCl and oxalic acid (5 mM) at indicated time points. (**d**) Western blot analysis to determine the expression of Oxdc after induction with HCl or Oxalic acid using rabbit polyclonal antibody raised against purified Oxdc. *F. velutipes* mycelia was induced by reducing the pH of the medium from 5.5 to 3.0 by adding oxalic acid (5 mM) or HCl. *F. velutipes* was grown at 23 °C and induced with oxalic acid for indicated time and concentration. Error bars represents standard error between three biological replicates. OA, oxalic acid.

With an aim to study the effect of oxalic acid on mycelial growth, *F. velutipes* was grown on potato dextrose agar (PDA) medium consisting of different concentrations of oxalic acid (1 mM, 5 mM and 10 mM). The growth of fungal mycelia was observed to be severely reduced with the increase in concentration of oxalic acid. At 1 mM, growth was almost comparable to normal PDA medium. Growth was comparatively less at 5 mM and no growth was observed in presence of 10 mM oxalic acid (Fig. 1a). Morphological response of fungi was also found to be different in presence of oxalic acid. Control mycelia spread loosely on oxalic acid free medium whereas dense mycelial growth/aggregate were observed on medium with oxalate (Fig. S1a,b). This is called phalanx growth strategy^18^ that probably is induced by accumulation of toxic metals in the medium due to chelating property of oxalic acid. We could not observe any growth of mycelia on low pH PDA medium (Fig. 1a) where HCl was used to adjust the pH equivalent to 5 mM oxalic acid (pH 3.0).

Since, Oxdc is an enzyme playing an important role in catabolism of oxalic acid; we have compared the expression of *OXDC* by quantitative real time- PCR in different conditions. *OXDC* expression was found to increase steadily with the increase in oxalic acid concentration from 1 mM to 10 mM (Fig. 1b). A nine fold higher expression of *OXDC* was observed at 10 mM oxalic acid in comparison to untreated control. Previously it was shown that *F. velutipes’ OXDC* gene is induced by acidic pH^17^. However, oxalic acid being the substrate for the enzyme should play some specific role in the induction of enzyme besides reducing the pH. Keeping this fact in mind, we compared the level of *OXDC* in the mycelium induced either by substrate oxalic acid (5 mM) or HCl (added to bring the pH of the medium to 3.0, equivalent to oxalic acid containing medium). Quantitative RT-PCR analysis revealed that though *OXDC* is induced in presence of both HCl and oxalic acid, but level of induction was 2-fold higher in presence of oxalic acid as compared to that of HCl (Fig. 1c). We have also performed Western blot analysis of total soluble protein extracted from *F. velutipes* with polyclonal antibody against Oxdc (Fig. 1d). The result correlated well with qRT-PCR data.

Cell’s internal homeostasis machinery can effectively get rid of excess oxalic acid by induction of oxalate catabolising enzymes like Oxdc which can reduce oxalic acid toxicity by degrading oxalic acid into formic acid and CO_2_. However, if we co-relate mycelial growth with *OXDC* expression data, it can be concluded that high level of *OXDC* can become limiting factor for fungal growth. In spite of striking increase in *OXDC* level associated with increased oxalic acid concentration; there is severe reduction in the growth of mycelia. This could be due to the fact that oxalic acid, at very concentration leads to many folds increase in *OXDC* level but the downstream enzymes may not be available in equally sufficient levels to act. This can lead to accumulation of toxic degradation products which cannot be metabolized further causing toxicity and growth inhibition. Lack of growth of *F. velutipes* in presence of HCl shows that unlike that of oxalic acid the fungus does not have the machinery to overcome the effects of low pH created by an inorganic acid like HCl.

### Fv*CaMLP* is an EF hand protein that binds the *OXDC* promoter

**Figure 2:**
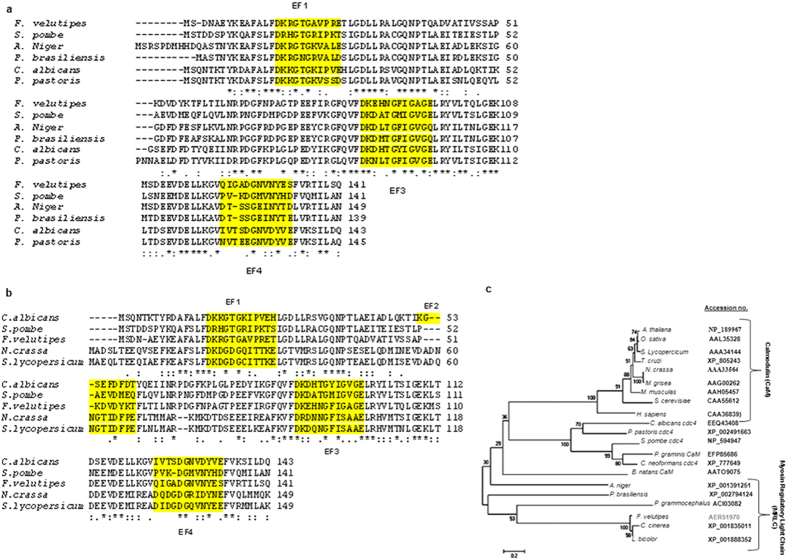
FvCamlp is an EF hand protein similar to Cdc4 and calmodulins. (a) Multiple sequence alignment of the deduced amino acid sequence of Fv*CaMLP* with myosin light chain (Cdc4p) from other organisms. (**b**) Comparison of EF hand motifs of FvCamlp with calmodulins and Cdc4p from other organisms by multiple alignment. Sequences were aligned using CLUSTAL W software (EBI). The putative calcium binding EF hand motifs (EF1, EF2, EF3 and EF4) are highlighted in yellow. (**c**) Phylogenetic tree illustrating the evolutionary relationship between FvCamlp, calmodulins and myosin light chain (Cdc4p) from different organisms. Sequences were taken from NCBI database and tree was constructed by neighborhood joining method using MEGA4 software. Accession numbers are provided in brackets. Values at the branch nodes indicate the numbers of trials out of 100 that produced each node. The 0.2 scale represents 20% change. CaM, calmodulin.

The above observations prompted us to identify the regulatory mechanism of *OXDC* upon induction by oxalic acid. With an aim to identify the proteins that bind to the *OXDC* promoter, we have performed yeast one hybrid assay. For this, 100 bp of the promoter region (−183 to −282) that included LPRE^17^ was used as bait. An EF hand protein showing similarity with myosin light chains and calmodulins (FvCamlp) was identified as a major *OXDC* promoter-interacting protein *in vivo*. The 426 bp of complete cDNA of Fv*CaMLP* encoding the protein of 141 amino acids was identified in yeast one hybrid screening. Multiple alignment of this putative calcium binding protein with myosin light chain-Cdc4 from other organisms revealed significant homology (Fig. 2a). It is 60% identical to myosin light chain -Cdc4 of *S. pombe*, 59% identical to Cdc4 of *C. albicans* and 47% identical to the calmodulin from *Solanum lycopersicum*. Multiple sequence alignment of FvCamlp with calmodulins and Cdc4 from other organisms (Fig. 2b) revealed that unlike calmodulins, which have four functional EF hand motifs and each chelating one Ca^2+^ ion^19^, FvCamlp has three potential EF hand motifs (EF1, EF3 and EF4). Gly at 6^th^ position was well conserved in the three EF hand motifs (EF1, EF3 and EF4), but Glu at the end of the loop was present only in EF1 and EF3 motifs. The second EF hand motif (EF2) in FvCamlp, consists of a four amino acid deletion and two amino acid insertion, a feature similar to other known Cdc4. Phylogenetic analysis showed FvCamlp to be evolutionarily close to myosin light chain from different organisms, including other basidomycetes like *Laccaria bicolor and Coprinopsis cinerea* (Fig. 2c). FvCamlp is also closely related to calmodulins from *Bigelowiella natans* and phytopathogen *Puccinia graminis.* The nucleotide sequence of Fv*CaMLP* and its 5′ flanking region, together with the amino acid sequence of the encoded polypeptide is shown in Fig. S2a.

### Effect of oxalic acid on Fv*CaMLP* expression

**Figure 3:**
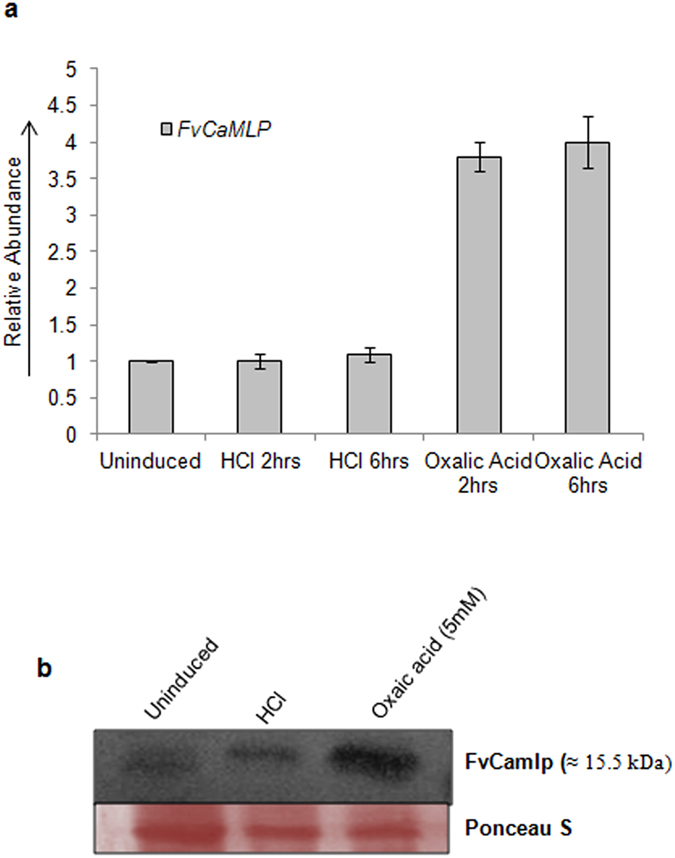
Expression analysis of Fv*CaMLP*. (**a**) qRT-PCR analysis to check the expression of Fv*CaMLP* in presence of oxalic acid or HCl. (**b**) Western blot analysis to determine the expression of FvCamlp after induction with HCl or oxalic acid using rabbit polyclonal antibody raised against purified FvCamlp. *F. velutipes* mycelia was induced by reducing the pH of the medium from 5.5 to 3.0 by adding oxalic acid (5 mM) or HCl. Error bars represents standard error between three biological replicates.

Since *OXDC* can be induced by low pH condition created by both oxalic acid and HCl, it was obvious for us to check the expression of Fv*CaMLP* transcript in presence of extracellular oxalic acid or HCl. qRT-PCR analysis showed around 4-fold upregulation of Fv*CaMLP* in response to oxalic acid (Fig. 3a). Interestingly, no significant change was observed in the basal level expression of Fv*CaMLP* in presence of low pH (HCl) as compared to uninduced control. In accordance with this data, Western blot analysis (Fig. 3b) using polyclonal antibody against FvCamlp raised in rabbit also showed more FvCamlp accumulation in presence of oxalic acid than HCl and uninduced control mycelia. Thus, Fv*CaMLP* is specifically induced by oxalic acid and not by low pH.

### FvCamlp is a calmodulin like Ca^2+^ binding protein

**Figure 4:**
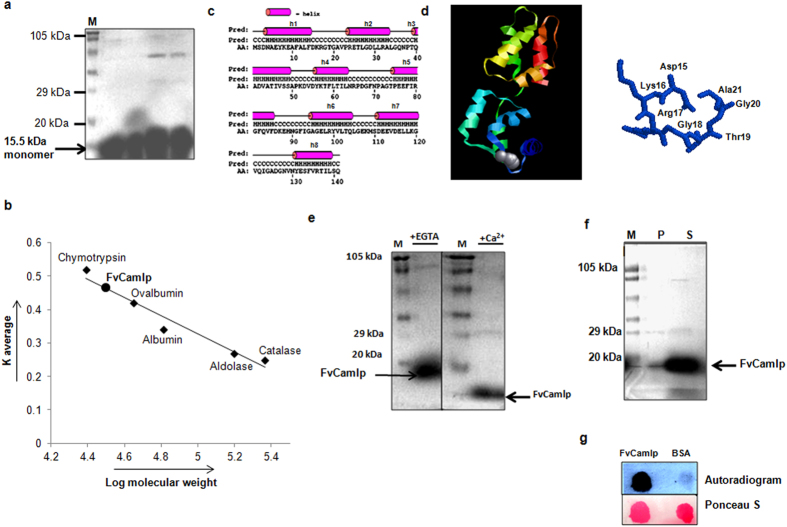
Purified FvCamlp binds Ca^2+^ and shows properties typical of calmodulin. FvCamlp expressed in *E. coli* BL21strain transformed with the pGST-Fv*CaMLP* was purified using glutathione affinity purification. (**a**) Purified FvCamlp in 12.5% SDS PAGE. (**b**) Calibration curve for the determination of FvCamlp molecular weight at pH 7.5 by gel filtration chromatography on Superdex 200 column. Marker proteins used for calibration: Chymotrypsin (25 kDa); Ovalbumin (45 kDa); Albumin (66 kDa); Aldolase (156.8 kDa); Catalase (232 kDa); Expected Molecular weight of FvCamlp (32 kDa). (**c**) The secondary structure prediction of the FvCamlp polypeptide using PSIpred V2.0 software. (**d**) Structure of FvCamlp predicted by the Phyre protein structure prediction tool. The predicted calcium ion binding site and the residues involved are also shown. (**e**) Ca^2+^-dependent electrophoretic migration shift of purified FvCamlp in 15% SDS PAGE with 5 mM EGTA (left) and 5 mM CaCl_2_ (right). (**f**) Heat stability of FvCamlp. The buffer containing purified FvCamlp was heated for 5 min at 95 °C, followed by centrifugation to pellet heat denatured protein from soluble heat-stable protein. FvCamlp was quantitatively recovered in the heat-stable supernatant fraction as observed on 12.5% SDS-PAGE. (**g**) Calcium^45^ overlay assay to show Ca^2+^ binding by FvCamlp. 20 μg of purified FvCamlp and BSA were blotted on nylon membrane and incubated in presence of calcium overlay buffer containing 1 μCi of Ca^45^Cl_2_. When blot was exposed to X-ray film, FvCamlp gave strong signal whereas bovine serum albumin (BSA) used as a negative control showed no signal. Ponceau S staining was done to confirm proper spotting of protein on nylon membrane. P, heat labile pellet; S, heat stable supernatant fraction; M, pre-stained molecular weight marker.

With an aim to purify FvCamlp for further characterization, complete cDNA of Fv*CaMLP* was cloned in IPTG inducible bacterial expression vector pGEX4T-2 and the resulting construct pGST-Fv*CaMLP* was transformed into BL21 strain of *E. coli.* Purified FvCamlp-GST fusion protein was subjected to thrombin cleavage to release FvCamlp free of GST tag. Purified FvCamlp thus obtained, migrated on 12.5% SDS PAGE with an apparent molecular weight of ≈15.5 kDa (Fig. 4a). In order to determine the molecular weight of the protein in native/non-denatured state, gel filtration/size exclusion chromatography was performed using Superdex-200 column. The elution volume of the protein was 15.49 ml (Kav- 0.468), which corresponds to ≈31 kDa molecular weight (Fig. 4b). This suggests that FvCamlp exist as a dimer under native conditions. Dimer formation of FvCamlp was independent of Ca^2+^ since it existed in dimeric state both in absence (5.0 mM EGTA) and presence of Ca^2+^ (5.0 mM).

Being an EF hand protein, *in silico* structure prediction of FvCamlp was done to predict the putative Ca^2+^ binding site. The secondary structure prediction of the FvCamlp polypeptide revealed the presence of eight alpha-helix interspersed between coil regions (Fig. 4c). A model was predicted for FvCamlp using Phyre (www.sbg.bio.ic.ac.uk/phyre/) remote homology prediction server (Fig. 4d). Ramachandran plot analysis of predicted model showed that 94.8% of the amino acid residues of the protein occupied most favorable region, 5.2% in additionally allowed region of the plot and none of the residues occupied the disallowed region. A functional calcium binding site was predicted in the model involving the residues Asp 15, Lys 16, Arg 17, Gly 18, Thr 19, Gly 20, and Ala 21 which form part of EF hand motif. When deduced amino acid sequence of FvCamlp was subjected to 3D-partner tool^20^ to predict its interacting partners, we got myosin heavy chain as well as serine/threonine-protein phosphatase 2B catalytic subunit (calcineurin) from *S. cerevisiae* and *S. pombe* as major hits (Table S2). Besides, *in silico* analysis of FvCamlp also predicted few Ser/Thr phosphorylation sites (Fig. S3) which can act as potential target for action of protein kinase. With an aim to find out the interacting partners of FvCamlp, we have performed GST pull down assay with FvCamlp-GST fusion protein. SDS- PAGE analysis showed the presence of three interacting proteins (Fig. S4). One of the proteins showing significant similarity to serine/threonine protein kinase of *Aspergillus clavatus* (gi|121710522) was identified by MALDI analysis (Table S4) of the excised protein bands. Other two proteins were found to be hypothetical.

Despite the divergence in sequence and structure, FvCamlp showed few properties typical of calmodulins. Minor but distinct calcium dependent alteration was observed in electrophoretic mobility of FvCamlp on 15% SDS PAGE, a characteristic of calmodulin and other members of EF hand calcium binding protein family. Purified FvCamlp migrated with an apparent molecular weight of ≈15.5 kDa in presence of added CaCl_2_ (5 mM) and was slightly retarded in migration in presence of added EGTA (5 mM) (Fig. 4e). Besides, in addition to its apparent calcium-binding properties, FvCamlp expressed in *E. coli* was found to be heat stable, a property typical of calmodulins and other members of the calcium-modulated family of proteins^21^. After heat treatment, most of the FvCamlp was detected in the heat stable supernatant fraction on the SDS-PAGE (Fig. 4f). We investigated calcium binding ability of FvCamlp by a direct and qualitative dot-blot calcium overlay method^22^. The purified FvCamlp blotted on nylon membrane was incubated in presence of calcium overlay buffer containing 1 μCi of Ca^45^Cl_2._ When blot was exposed to X-ray film, FvCamlp gave strong signal whereas bovine serum albumin (BSA) used as a negative control showed no signal (Fig. 4g).

### FvCamlp binds *OXDC* promoter in presence of Ca^2+^

**Figure 5:**
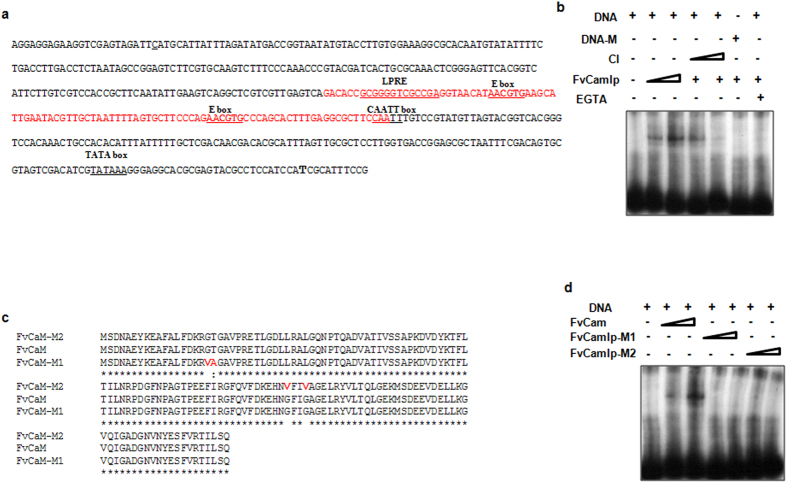
FvCamlp binds to the *OXDC* promoter. (**a**) Nucleotide sequence of the 5′ upstream region of *OXDC* gene. 100 bp of sequence (−183 bp to −282 bp) consisting of E box elements and LPRE (Low pH response element) used as probe in electrophoretic mobility shift assay (EMSA) is depicted in red. (**b**) EMSA with purified FvCamlp using γP^32^ATP labeled 100 bp of *OXDC* promoter as probe. In lane 2, 500 ng and from lane 3–7, 1.0 μg of purified FvCamlp was used. For competitive inhibition (CI) in lane 4 and 5 unlabelled E box element was added in 50 and 100 molar excess respectively. In lane 6 γP^32^ATP labeled 100 bp of *OXDC* promoter with mutated E box element was used as probe. In lane 7 gel shift assay was performed in presence of 5 mM EGTA. Triangle represents increasing amount of either protein or DNA. (**c**) Amino acid sequences of FvCamlp and site-directed mutagenesis products of FvCamlp (FvCamlp-M1 and FvCamlp-M2) are shown. The amino acid substitutions are shown in red. (**d**) Gel shift assay showing that FvCamlp, but not FvCamlp-M1 and FvCamlp-M2, is able to bind to γP^32^ATP labelled 100 bp *OXDC* promoter. Triangle represents increasing concentration (500 ng and 1 μg respectively) of indicated protein used for gel shift assay.

*In silico* analysis of 100 bp fragment (−183 to −282) from *OXDC* promoter used as bait in yeast one hybrid assay, revealed the presence of two non-canonical E-box elements (AACGTG) (Fig. 5a). E-box elements are 6-bp DNA elements with consensus sequence of CANNTG^23^. However, there exist other E-boxes of similar sequences called non-canonical E-boxes. These are mainly recognized by bHLH (basic helix loop helix) family transcription factors. Since topology of EF-hand motif of FvCamlp is similar to the helix-turn-helix DNA binding domain, EMSA was performed with purified FvCamlp expressed in *E. coli*. We observed that FvCamlp could interact efficiently with a γP^32^ATP labelled 100 bp fragment (−183 to −282) from *OXDC* promoter (Fig. 5b). We have also performed competitive EMSA with excess of unlabelled non-canonical E box DNA element. Unlabelled E box element could efficiently compete with labelled probe for binding with FvCamlp, hence ensuring the specificity of the complex. Furthermore, no binding was observed when γP^32^ATP labeled 100 bp of *OXDC* promoter with mutated E box element (site directed mutagenesis to substitute AACGTG with AGCATG) was used as probe. DNA binding ability of FvCamlp was also determined in presence of EGTA which chelates the Ca^2+^ ions. A significant reduction in DNA binding ability of FvCamlp was observed in presence of 5.0 mM EGTA (Fig. 5b). Binding of Ca^2+^ ions to the protein might lead to certain conformational changes allowing it to interact with the DNA. These results show that FvCamlp binds specifically to the E box element of *OXDC* promoter in presence of Ca^2+^ ions.

The EF-hand motif contains a helix-loop-helix topology (alpha helices linked by a short loop region of about 12 amino acids) similar to many DNA binding proteins^24^. So, we performed site directed mutagenesis in EF- hand motifs of FvCamlp (FvCamlp-M1 and FvCamlp-M2) to confirm their role in DNA binding. It has been shown previously that substitution of even a single amino acid in the EF hand could alter the target specificity of calmodulin^25^. In FvCamlp-M1 Gly and Thr residues of EF1motif were substituted by Val and ala respectively and in FvCamlp-M2 two glycine residues of EF3 motif were substituted by valine (Fig. 5c). EMSA with purified mutated FvCamlp-M1and FvCamlp-M2 showed that these proteins have lost the ability to bind the *OXDC* promoter (Fig. 5d). This suggests that EF hand motifs are required for the DNA binding ability of FvCamlp.

### Sub-cellular localization of FvCamlp

**Figure 6:**
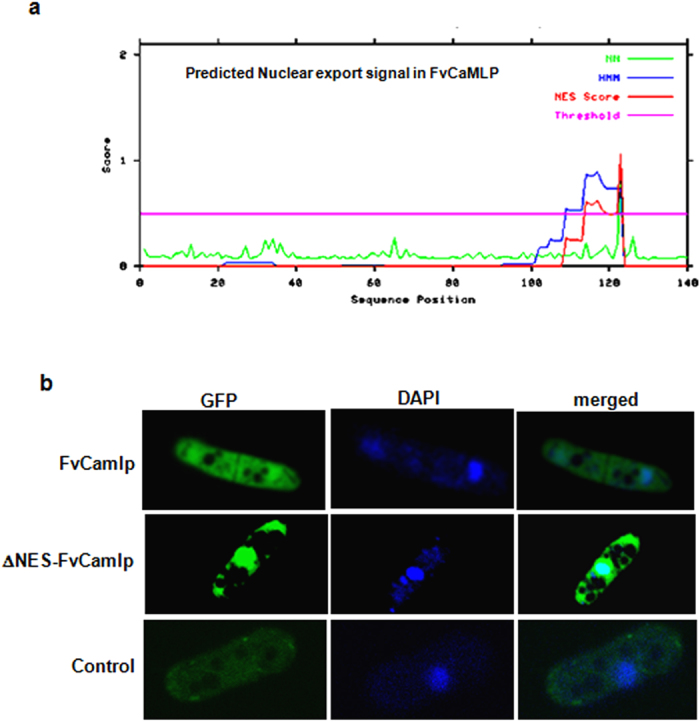
Sub-cellular localization of FvCamlp. (**a**) Prediction of nuclear export signal in FvCamlp using the Expasy tool Net NES1.1 which predicts leucine-rich nuclear export signals (NES) in eukaryotic proteins through a combination of neural networks and hidden Markov models. (**b**) Confocal microscopy of *S. pombe* cells expressing: GFP tagged Fv*CaMLP* cloned in pTN54 vector; *S. pombe* cells expressing GFP tagged Fv*CaMLP*ΔNES cloned in pTN54 vector; Empty pTN54 vector. *S. pombe* cells were grown to early logarithmic phase and prepared for florescence microscopy. DAPI staining of the nucleus was performed to confirm the nuclear localization of FvCamlp.

In order to bind *OXDC* promoter, FvCamlp must enter the nucleus. Nucleo-cytoplasmic trafficking of a protein through nuclear pore complex by active transport requires both nuclear localization signal (NLS) as well as nuclear export signal (NES). No known conventional nuclear localization signal (NLS) motif could be predicted by *in silico* analysis of FvCamlp protein. NES is a short sequence of 4 hydrophobic amino acid residues (L_xxx_L_xx_L_x_L, where “L” is a hydrophobic residue (often leucine) and “x” is any other amino acid) in a protein that targets its export from the nucleus to the cytoplasm. A putative leucine rich NES (nuclear export signal) was predicted near to C terminus of FvCamlp (VDELLKGVQI) (Fig. 6a).

We have chosen fission yeast *S. pombe* (BJ7468 strain) as a heterologous system to determine the sub-cellular localization of FvCamlp. Full length cDNA of Fv*CaMLP* and mutated cDNA Fv*CaMLP*ΔNES (−NES) were cloned in expression vector pTN54 with GFP fused at N terminus under thiamine repressible nmt1promoter. In *S. pombe* strain expressing FvCamlp-GFP fusion protein, the GFP fluorescence was uniformly distributed throughout the cytosol and significant fluorescence was also observed in the nucleus (Fig. 6b). However, strain expressing Fv*CaMLP*ΔNES –GFP showed a much higher GFP fluorescence in the nucleus as compared to that of the cytosol, which was confirmed by DAPI staining. This could be due to accumulation of fusion protein in the nucleus in the absence of transport to cytoplasm. Uniform florescence was observed in yeast strain expressing empty vector (Fig. 6b). Moreover, unlike cdc4 of *S. pomb*e, FvCamlp was not localized in the medial region of the dividing cell at the contractile ring^8^. Thus, nuclear localisation and interaction of FvCamlp with *OXDC* promoter suggests its role in the expression of *OXDC*.

### siRNA mediated suppression of Fv*CaMLP* leads to transcriptional down regulation of *OXDC* expression

**Figure 7:**
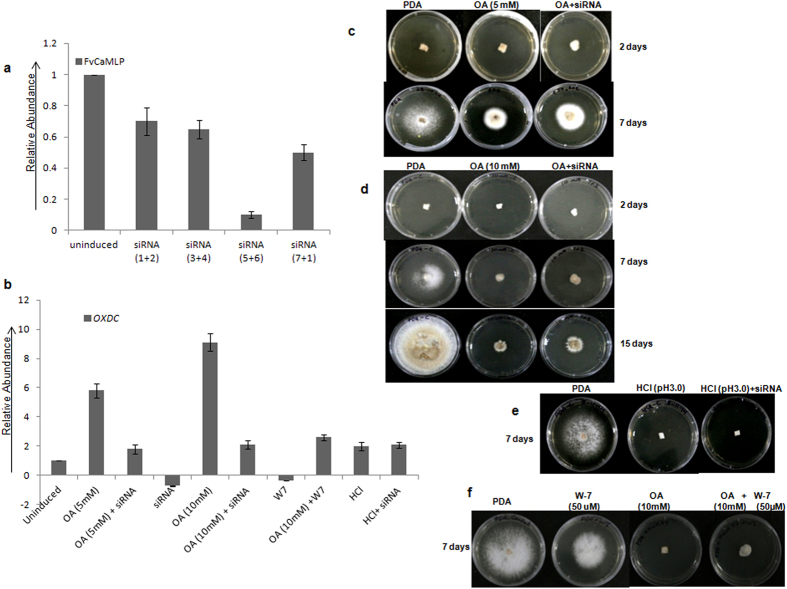
Effect of siRNA mediated Fv*CaMLP* suppression on *OXDC* expression and *F. velutipes* growth. (**a**) Expression analysis of Fv*CaMLP* in siRNA treated fungal mycelia. Fungal mycelia were grown in liquid culture media with four different combinations of siRNAs (1 + 2; 3 + 4; 5 + 6 & 7 + 1, 25 nmoles each) along with and without oxalic acid for 12 hours. Error bars represents standard error between three biological replicates. (**b**) Expression analysis of *OXDC* in siRNA treated fungal mycelia under indicated conditions. 25 nmoles each of siRNA 5 + 6 combination was used for silencing. 50 μM W-7 was used for the analysis. HCl was added to bring the pH to 3.0 equivalent to 5 mM oxalic acid. (**c**) Growth of *F. velutipes* mycelia on PDA in presence of 5 mM oxalic acid. (**d**)10 mM oxalic acid. (**e**) Inorganic acid HCl (pH 3.0). (**f**) Calmodulin antagonist W-7 hydrochloride (50 μM). Radial growth was monitored after 2 days, 7.0 days and 15 days respectively. OA, Oxalic acid.

To further understand the role of FvCamlp in the regulation of *OXDC* expression, *FvCaMLP* was suppressed through RNAi strategy using synthetic siRNAs (Table S3) targeting Fv*CaMLP* mRNA. For silencing, fungal mycelia grown in liquid culture media^17^ was treated with four different combinations of siRNAs (1 + 2; 3 + 4; 5 + 6 & 7 + 1, 25 nmoles each) in presence or absence of oxalic acid for 12 hours. Quantitative RT-PCR analysis of mycelia treated with different synthetic siRNA combinations showed significant reduction in Fv*CaMLP* transcript (Fig. 7a). Further, siRNA combination (5 + 6) which produced maximum suppression of Fv*CaMLP* transcript was used to analyze *OXDC* expression (Fig. 7b). Expression of *OXDC* was also found to be reduced significantly upon siRNA mediated suppression of FvC*aMLP* both at 5 mM and 10 mM oxalic acid. Even basal level of *OXDC* expression was found to be reduced in uninduced (without oxalic acid induction) mycelia treated with siRNA. This observation shows that *OXDC* is positively regulated by Fv*CaMLP* at transcriptional level. However, the level of *OXDC* transcript induced in presence of HCl remained unaffected by siRNA mediated suppression of *FvCaMLP* (Fig. 7b).

The fact that FvCamlp is a Ca^2+^ sensor prompted us to investigate if calmodulin mediated calcium signaling plays any role in *OXDC* transcription. We exposed *F. velutipes’* mycelium to 50 μM of N-(6-aminohexyl)-5-chloro-1-naphthalenesulfonamide hydrochloride (W-7 hydrochloride) along with oxalic acid. W-7 is a known calmodulin antagonist which binds calcium-loaded calmodulin (Ca^2+^- CaM) and blocks the Ca^2+^ signal messenger function of CaM^26^. In presence of W-7 inhibitor, *OXDC* expression was notably reduced both in presence and absence of oxalic acid (Fig. 7b) showing role of calmodulin mediated Ca^2+^ calcium signalling in *OXDC* expression.

In order to observe the affect of Fv*CaMLP* suppression on mycelial growth, we have grown fungal mycelia on PDA medium (consisting of 5 mM or 10 mM oxalic acid) in presence of siRNAs targeting Fv*CaMLP* mRNA. Radial growth of mycelia was monitored regularly. Interestingly, growth of mycelia on PDA with only oxalic acid (5 mM or 10 mM) was slow as compared to PDA consisting of synthetic siRNAs (5 + 6) along with oxalic acid (Fig. 7c,d). Thus, Fv*CaMLP* suppression promoted mycelial growth in presence of oxalic acid. Fungal mycelia grow normally on PDA plates without oxalic acid used as control. Mycelial growth was unaffected when mycelia was grown on normal PDA medium (-oxalic acid) consisting of siRNA (Fig. S1c). This shows that suppression of Fv*CaMLP* does not affect the growth of mycelia under normal condition. However, when same experiment was repeated on low pH medium (using HCl), the growth was not promoted by suppression of Fv*CaMLP* with siRNA (Fig. 7e). This confirms the oxalic acid specific response of Fv*CaMLP*. The mycelial spread was less dense on addition of siRNA to oxalic acid containing PDA compared to mycelia grown only in presence of oxalic acid (Fig. S1b). Interestingly more growth was observed in presence of W-7 hydrochloride (50 μM) in PDA consisting of 10 mM of oxalic acid compared to plate consisting of only 10 mM of oxalic acid (Fig. 7f). This suggests that inhibition of calmodulin promotes mycelial growth in presence of oxalic acid. Growth of mycelia was slow in presence of PDA (−oxalic acid) medium with only W-7. This could be due to the fact that inhibition of calmodulin might affect pathways needed for normal growth.

These results suggest that downregulation of *OXDC* either by siRNA mediated Fv*CaMLP* suppression or calmodulin inhibition, both promotes growth in presence of oxalic acid. This could be because of comparatively less accumulation of toxic degradation products of oxalic due to reduced level of Oxdc in either condition.

## Discussion

Wood rotting fungi secrete oxalic acid for the degradation and conversion of wood lignin and carbohydrate polymers. Since, oxalic acid is a toxic compound regulation of intracellular and extracellular concentration is imperative. *F. velutipes*, a wood rotting fungus responds to oxalic acid by induction of an oxalate degrading enzyme, oxalate decarboxylase. Oxdc exists as hexamer and Mn^2+^ is critical for its substrate recognition and catalysis^27^. It is assumed that the major role of Oxdc in fungi is to control the intracellular levels of oxalic acid, and thereby to regulate excess secretion of oxalic acid^28^,^29^. Secondly, Oxdc is required to keep steady levels of pH and oxalate anions outside the fungal hyphae, as supplementation of oxalic acid or a change to more acidic environmental pH levels often promotes Oxdc activities^16^,^28^,^29^,^30^,^31^.

In an attempt to understand the oxalic acid induced expression of *OXDC*, we identified a Ca^2+^ binding EF hand protein- FvCamlp which interacts with the E box elements present in *OXDC* promoter. FvCamlp can be considered to belong to a new class of EF hand calcium binding protein. It shows significant homology to both myosin light chain- Cdc4 and CaMs, but has properties typical of calmodulin like resistance to heat and Ca^2+^ dependent mobility shift. Besides, gene organization of Fv*CaMLP* is also similar to most fungal calmodulins with five introns and location of first intron immediately after ATG (supplementary information). Like CaMs, FvCamlp also has a functional calcium binding site as shown by Ca^2+^ overlay assay. Cdc4 does not binds calcium *in vitro*^32^. Gel filtration chromatography shows FvCamlp exists ain dimeric form as known in calmodulins^33^. However structure prediction of Cdc4 (*S. pombe*), the most well characterized protein of this category in yeast/fungal system shows that cdc4 exists as a monomer^32^. Localization studies also show the difference in behaviour of FvCamlp from Cdc4. Unlike Cdc4 of *S. pombe* which was reported to localize to contractile ring at medial region of the cell during cytokinesis^8^, FvCamlp was uniformly distributed in nucleus and cytoplasm like CaMs^34^. In the absence of obvious NLS, nuclear entry of FvCamlp can be explained by the fact that being small in size, it might enter the nucleus by traversing the NPC (nuclear pore complex) by passive diffusion or may form complex with other protein with NLS. It may have an unconventional NLS which needs to be identified. Its putative nuclear export signal (NES) might be involved in nucleo-cytoplasmic shuttling since its deletion lead to nuclear accumulation of FvCamlp, hence suggesting that FvCamlp is dynamic between the two compartments. Thus, FvCaMLP can be considered a CaM like protein.

Our data shows that like *OXDC*, Fv*CaMLP* is also induced in presence of oxalic acid and is localised into the nucleus suggesting its involvement in the expression of oxalate decarboxylase. It is known that oxalic acid through acidification and via its chelating properties may increase the availability of metal ions like Ca^2+ ^35. Thus, presence of oxalic acid in the extracellular medium might result in change of cytosolic Ca^2+^ concentration, leading to activation of calcium signaling pathway. FvCamlp being a Ca^2+^ binding protein might act as one of the components of the pathway, which after entering the nucleus binds to *OXDC* promoter and promotes its transcription. The ability of FvCamlp to bind E box element can be explained by the fact that the topology of EF**-**hand motif of FvCamlp is quiet similar to the helix**-**turn**-**helix (bHLH) DNA binding domain of transcription factors that are known to recognize the E box element. This was further confirmed when DNA binding ability of FvCamlp was lost, when mutated versions of FvCamlp (site directed mutagenesis of various EF hand residues) were used. Besides, just as bHLH forms a dimerization domain that allows proteins to form homo- and/or heterodimers^36^, FvCamlp also exist as a dimer. FvCamlp binding site (non- canonical E box) lies in close proximity to LPRE, so it may modulate *OXDC* expression either directly or indirectly through modulating binding of LPRF.

Besides, it was also observed that in presence of calmodulin inhibitor (W-7) expression of *OXDC* was significantly reduced even in presence of functional Fv*CaMLP*. This suggests that Fv*CaMLP* acts downstream to calmodulin. FvCamlp was found to interact with a protein showing similarity to Ser/Thr protein kinase which might act on one of the predicted Ser/Thr phosphorylation residues in FvCamlp (Fig. S3). Moreover, calcineurin, a calcium/calmodulin-activated protein phosphatase has also been predicted to be a potential interacting partner of FvCamlp and this interaction might be necessary for the activation of FvCamlp through a dephosphorylation event of the predicted Ser/Thr phosphorylated residues (Fig. S3) followed by its nuclear translocation.

**Figure 8:**
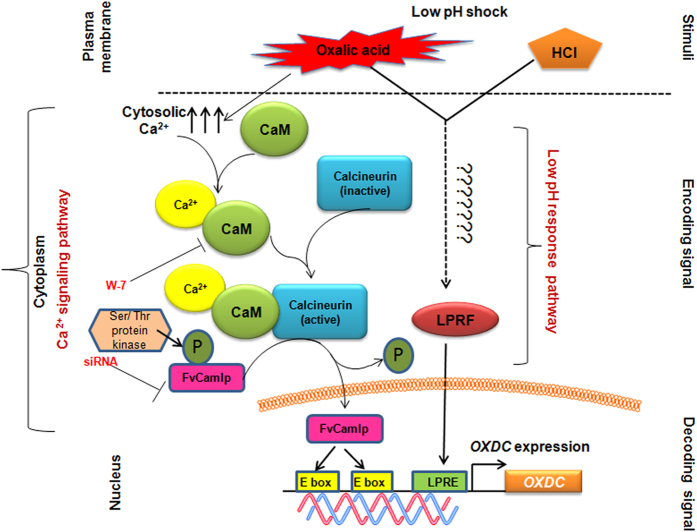
Proposed model for transcriptional regulation of oxalate decarboxylase. Oxalic acid, in the extracellular medium might result in the change in concentration of cytosolic Ca^2+^ that might activate FvCamlp *via* calmodulin/calcineurin mediated Ca^2+^ signaling pathway leading to *OXDC* transcription. Blocking of this signalling pathway either by use of calmodulin (CaM) inhibitor (W-7) or siRNA specific to Fv*CaMLP* leads to significant downregulation of *OXDC* in presence of oxalic acid. Besides oxalic acid also being an organic acid creates low pH stress, leading to activation of an additional low pH response pathway which might turn on the *OXDC* transcription through low pH response factor (LPRF). In presence of any other general acid like HCl, only low pH response pathway gets activated and calcium signaling pathway is probably not involved. LPRE, Low pH response element.

Changes to gene expression require a coordinated effort from multiple pathways in order to allow a limited amount of proteins to achieve the complicated feat of surviving unfavourable conditions. The presence of low pH responsive factor (LPRF) in both acid and oxalic acid induced cell extract^18^ shows that low pH response pathway probably gets activated in presence of both oxalic acid and HCl, since low pH is created in either condition. However, oxalic acid through its ability to modulate the availability of Ca^2+^ ions leads to activation Fv*CaMLP* which further induces the expression of *OXDC.* This assumption is further supported by the observation that though *OXDC* is induced by both oxalic acid and HCl, but there is 2–5 fold higher induction by oxalic acid as compared to HCl. This increase can be attributed to the activation of Ca^2+^ signaling pathway in addition to low pH response pathway in presence of oxalic acid. Based on this data, we have proposed a model for Ca^2+^/Calmodulin/Calcineurin and FvCamlp dependent signalling pathway for transcriptional regulation of *OXDC* (Fig. 8). Fv*CaMLP* can be considered as new class of unconventional DNA binding EF hand protein showing sequence similarity to both Cdc4 and CaMs. Though, BLAST analysis shows presence of Fv*CaMLP* homologs in many other fungi *Coprinopsis cinerea* (XP_001835011.2), *Phanerochaete carnosa* (XP_007397807.1), *Laccaria bicolor* (XP_001888352.1) etc. but none of them have been characterized. Based on this report, this protein can be studied in other organisms for similar kind of regulatory function.

## Methods

### Strains, media and growth conditions

*Flammulina velutipes* (ATCC13547) was maintained on potato dextrose agar or liquid media^17^ (5.0% dextrose, 1.0% peptone, 0.1% KH_2_PO_4_, 0.05% MgSO_4._7 H_2_O and 1% malt extract) at 23 °C for 15–30 days. *S. pombe* was grown aerobically at 30 °C in either rich media YES (0.5% yeast extract, 3% dextrose) or selective synthetic medium (EMM) as described elsewhere^37^. *S. cerevisiae* was grown on YPD (1.0% yeast extract, 2.0% peptone, 2.0% glucose) or SD medium (0.67% YNB w/o amino acids and 2.0% glucose). The *E. coli* DH5α strain (used as general cloning host) and BL21(DE3)strain (for recombinant FvCamlp protein expression) were grown in LB medium (Invitrogen) at 37 °C. Ampicillin (100 μg mL^−1^) and kanamycin (50 μg mL^−1^) were added to the medium when necessary. Genetic transformation of yeast was carried out by the alkaline cation method^38^. List of strains and plasmids used in the study is given in Table S1.

### Plasmid construction

100 bp of *OXDC* promoter was PCR amplified using a forward primer containing an *EcoRI* restriction site (5′-GAATTCGAC ACC GCG GGG TCG CCG AGG-3′) and reverse primer containing a *SpeI* restriction site (5′-ACTAGTTTG GAA GCG CCT CAA AGT GCT GGG CAC G-3′). The amplified DNA fragment was ligated to the *EcoRI* and *SpeI* sites of pHIS 2.1 vector (Clontech) yielding pHis-OxPro which was used as bait in yeast one hybrid screening. To obtain pGST- Fv*CaMLP,* cDNA of Fv*CaMLP* was amplified using the forward primer with *BamHI* site (5′-GGATCCATGAGCGACAACGCA GA AT ACAAAGAG-3′) and the reverse primer with *NotI* site (5′-GCGGCCGCTCATTGGC TGAGGATA GTGCGTAC-3′). The amplified fragment (426p) was cloned in *BamHI* and NotI restriction sites of the bacterial expression vector pGEX-4T2 (GE Biosciences). pGFP-Fv*CaMLP* and pGFP-Fv*CaMLP*ΔNES used for studying sub-cellular localization were constructed by amplifying cDNA of Fv*CaMLP* with forward primer containing *SalI* site (5′-CGGTCGACGAGCGACAACGCAGAATAC-3′) and reverse primer with *NotI* site (5′-GCGCGGCCGCTCATTGGCTGAGGATAGTG-3′). Fv*CaMLP*ΔNES was amplified with forward primer (5′-CGGTCGACGAGCG ACAACGCAGAATAC-3′) and reverse primer (5′-GCGCGGCCTCCTCGTCCGACATCTTCT C3.′Amplicons thus obtained were cloned in SalI and NotI sites of *S. pombe* expression vector pTN-54 (with leucine marker) in frame to N terminal GFP.

The nucleotide sequence of the Fv*C5SD* cDNA was determined from both strands by the double-strand dideoxy-chain terminator method by using an ABI3730xl DNA analyzer sequencer for automated sequence analysis.

### Yeast one hybrid

pHis-OxPro construct (with leucine marker) was used as bait for yeast one hybrid. The construct has a *HIS3* reporter which is expressed only upon interaction between a DNA-binding protein and the bait DNA. Yeast one hybrid assay was done using Matchmaker™ one hybrid library construction and screening kit from Clontech as per the manufacturer’s instructions. *F. velutipes’* mycelium was induced by oxalic acid (14 mM) for 6.0 hrs and mRNA was isolated using DynabeadsR mRNA purification kit (DYNAL biotech). The double stranded cDNA prepared from 1.0 μg of poly A^+^ RNA (by SMART™ cDNA synthesis technology) was used for generating the cDNA library. cDNA library expressed in fusion with Gal4 activation domain was generated by co-transformation of double stranded cDNA with linearized pGADT7- Rec2 plasmid (with tryptophan marker) and was used as prey for the assay. Bait construct, double stranded cDNA and linearized pGADT7- Rec2 plasmid were co- transformed in Y187 stain of *S. cerevisiae* and transformants were selected on minimal media (−His, −Leu, −Trp) with 3-AT (3-Amino-1,2,4-triazole) at concentration of 70 mM which was tested to be optimum for inhibiting basal level of His3 expression. Finally, 60 healthy yeast colonies were streaked and restreaked on minimal media with 100 mM 3-AT to eliminate false positives. Library plasmids were rescued from single healthy yeast colonies^39^ and transformed into *E. coli* strain DH5α followed by sequencing of isolated plasmids.

### qRT—PCR

15-day-old *F. velutipes* mycelia was either induced with HCl (to pH 3) or oxalic acid (5 or 10 mM) for desired time points and total RNA was isolated using Tripure reagent (Roche) according to manufacturer’s instructions. cDNA was synthesized from total RNA (5 μg) using an oligo dT primer (Invitrogen) and Superscript I reverse transcriptase (Invitrogen). The original mRNA template was removed from the RNA: DNA heteroduplex with RNase H. The cDNA samples were then used as templates for qRT-PCR in a 7900 Fast Real Time PCR system (Applied Biosystems) using SDS 2.4 program (Applied Biosystems). The reaction mix included 2X KAPA SYBR FAST (Kapa Biosystems), 0.5 μl of cDNA and 0.2 μM primers per 10 μl reaction. The amplification program included Stage I- 50 °C for 2 min, Stage 2- 95 °C for 10 min, Stage 3- 40 cycles of 95 °C for 15 sec and 60 °C for 60 sec, Stage 4- 95 °C for 15 sec, 60 °C for 15 sec and 95 for 15 sec. Raw data was analysed using RQ manager 1.2.1 software (Applied Biosystems) to determine relative gene expression. Values were normalized with the amplification of *GAPDH* as a constitutively expressed internal control. The results represent the average of three biological replicate experiments each performed in triplicates. Error bars, where they exist represent the standard error between 3 separate biological replicates. The following primers designed with Primer Express (V- 3.0) software (Applied Biosystems) were used for qPCR analysis. *GAPDH1*-F5′ TCCGAAGGTTCCCTCAAAGA 3′ & *GAPDH2*-R5′ GAAGATGGACGAGCGGTTGT 3′; Fv*CaMLP-*F5′TTCGACAAGGAGCACAATGG3′ and Fv*CaMLP-*R5′ CATCTTCTCGCCCA GTTGTGT3′; *OXDC*F -5′ ATACCGACCGTTTTGCTGATG 3′ and *OXDC*-R5′GCAAGTGT CTCGTCGTCCAA3′.

### Protein extraction and Western blotting

Total soluble protein extraction from *F. velutipes* mycelium was carried out as described in^17^. 20 μg of Crude protein extract was subjected to 12.5% SDS-PAGE^40^ and blotted onto nitrocellulose membrane (Amersham-Pharmacia) by electro transfer^41^. The Oxdc and FvCamlp were detected by immunostaining with rabbit polyclonal antibody raised against Oxdc and FvCamlp respectively.

### Expression and purification of GST tagged protein

BL21 (DE) strain of *E. coli* was transformed with pGST-Fv*CaMLP* construct. Ampicillin resistant cells were grown at 37 °C to an O.D_600_ _nm_ of 0.5 followed by induction with 0.3 mM IPTG for 4 hrs. Cells were harvested by centrifugation at 5500 rpm at 4 °C for 15 min. Cells were washed twice with 1 X PBS. Pellet was resuspended in protein extraction buffer (1 mM DTT, 1 mM PMSF, 1 mM EDTA (pH 8.0) in 1XPBS pH 7.4). Lysozyme was added to the concentration of 1 mg/ml and incubated on ice for 30 min. with occasional mixing in between. Triton X 100 (0. 2%) and DNase (5 μg/ml) were added to the above mix followed by vigorous shaking. Incubation was done for 1 hr at 4 °C with gentle shaking to solubilize the fusion protein. It was centrifuged at 5000 rpm for 30 min. The above supernatant was clarified by filtration through 0.4 μ filters.

Large scale batch purification of GST tagged protein was done using 50% slurry of glutathione-sepharose resin (GE biosciences). Fusion protein (≈43 kDa) was cleaved of GST tag using thrombin (Sigma) at concentration of 10 units/mg protein to obtain purified FvCamlp with molecular weight of ≈15.5 kDa.

### Molecular weight determination of FvCamlp

Molecular weight of FvCamlp was estimated by ACTAFPLC (GE) using a pre-equilibrated Superdex 200 column (GE) 2.6 × 60 cm (Total volume-24 ml; Void volume −8 ml). Buffer consisting of 25 mM Tris-HCl pH 7.5 and 150 mM NaCl at 4 °C was used for the analysis (Flow rate- 0.4 ml/min). Standard proteins used for this analysis were Chymotrypsin (25 kDa); Ovalbumin (45 kDa); Albumin (66 kDa); Aldolase (156.8 kDa); Catalase (232 kDa). Partition coefficient (Kav) was calculated by using the formula: Kav = Ve−Vo/Vt−Vo. Ve-elution volume; Vo-Void volume; Vt- tolal volume.

### Electrophoretic mobility shift assay (EMSA)

Radiolabelled probe for EMSA were prepared by end labelling with [γ-P^32^] ATP (3000 Ci/mmol, Perkin Elmer), using T4 polynucleotide kinase (New England Biolabs), and separated from free nucleotide by a sephadex G-50 spin column. [γ-P^32^] ATP labelled probe was incubated with purified FvCamlp in 5X binding buffer (20% glycerol, 5 mM MgCl2, 2.5 mM EDTA, 2.5 mM DTT, 250 mM NaCl, 50 mM Tris-HCl (pH 7.5), 0. 25 mg/ml of poly dA-dT) at room temperature for 30 mins. Protein-DNA complexes and free DNA were fractionated on 5% polyacrylamide gels in 1xTBE buffer pH 8.3, at 4 °C and visualized by autoradiography.

### ^45^Ca overlay assay

The assay was performed as described in^25^ with minor modifications. 10 μg of purified FvCamlp was spotted on a nitrocellulose paper and washed with buffer containing 20 mM Tris (pH 7.0) and 50 mM KCl. The blot was incubated in the same buffer containing 1 μCi ^45^Ca (PerkinElmer Life Sciences) for 10 min at room temperature and then washed three times with 50% ethanol, air-dried, and exposed to X-ray film.

### Sub-cellular localization

pGFP-Fv*CaMLP* and pGFP-ΔFv*CaMLP*ΔNES construct were transformed into *S. pombe* and transformants were selected on SD minimal media without leucine. For expression of GFP tagged protein, transformants were grown in EMM medium till early log phase. After washing twice in 1x PBS pH 7.4, DNA was counterstained with DAPI (4′, 6-diamidino-2-phenylindole) and visualized in confocal microscope (Model No. TCS-SP2; Leica Microsystems, Germany) using appropriate set of filters.

### Bioinformatics analysis

BLAST database^42^ searches were performed using the National Center for Biotechnology Information (NCBI) server (http://www.ncbi.nlm.nih.gov/BLAST/). Clustal W software^43^ was used for multiple sequence alignment and Molecular Evolutionary Genetics Analysis (MEGA) software version 4.0^44^ was used for construction of phylogenetic tree. *In silico* analysis for detection of cis-regulatory element was done by TFsitescan^45^. Structure prediction was done by Phyre, remote homology modeling server^46^ and validation was done by procheck analysis^47^. Secondary structure prediction was done by PSIpred V2.0 using neural networks. NES (nuclear export signal) was detected by NetNES1.1 (http://www.cbs.dtu.dk/services/NetNES1.1/index.php). Prediction of interacting partners of the protein was done by BioXGEM 3D-partner server^23^.

### Treatment of *F. velutipes* mycelia with siRNA

The coding sequence (426 bp) of FvCaMLP was subjected to an algorithm developed by Bingbing Yuan^48^ *et al.* to identify the siRNA candidates. SiRNA were scored as previously described^49^ to find out the best set of siRNA with high silencing potency, target specificity and minimum off target binding. SiRNAs with score ≥7 were chosen for synthesis by Ambion (life technologies). List of siRNA used in the study is given in Table S3.

*F. velutipes* mycelia were treated with synthetic siRNA combination by the procedure previously described^50^,^51^ with necessary modifications. Mycelia of *F. velutipes* were grown in the liquid medium ((5.0% dextrose, 1.0% peptone, 0.1% KH2PO4, 0.05% MgSO4.7H2O and 1% malt extract) at 23 °C for 10 days. For RT- PCR analysis, siRNAs combinations were added to the liquid medium (at a final concentration of 25 nmole each) for another 12 hours. Later, mycelia were harvested and stored at −80 °C until RNA extraction. For radial growth assay siRNAs (25 nmole each) were plated on the surface of potato dextrose Agar (PDA) medium and mycelial growth was monitored after 48 hours and one week.

## Additional Information

**Accession Numbers:** Sequence data of FvCaMLP gene has been submitted to GenBank databases under accession number AER51970.

**How to cite this article**: Kamthan, A. *et al.* A calmodulin like EF hand protein positively regulates oxalate decarboxylase expression by interacting with E-box elements of the promoter. *Sci. Rep.* **5**, 14578; doi: 10.1038/srep14578 (2015).

## Supplementary Material

Supplementary Information
