# Supplementary material for: A calmodulin like EF hand protein positively regulates oxalate decarboxylase expression by interacting with E-box elements of the promoter
Source: Sci Rep. 2015 Oct 12;5:14578. doi: 10.1038/srep14578 (PMC4600981; doi:10.1038/srep14578)

<u>**Supplementary information**</u>

A calmodulin like EF hand protein positively regulates oxalate decarboxylase expression by interacting with E-box elements of the promoter.

Ayushi Kamthan[1#], Mohan Kamthan[2#], Avinash Kumar[1], Pratima Sharma[1], Sekhu Ansari [1], Sarjeet Singh Thakur[1], Abira Chaudhuri[1] and Asis Datta[1]∗

[1]National Institute of Plant Genome Research, New Delhi, India.

[2] Present address- Indian Institute of toxicology Research, Lucknow- 226 001

#Authors contributed equally to this work

∗To whom correspondence should be addressed: National Institute of Plant Genome Research, Aruna Asaf Ali Marg, New Delhi- 110067, India.

Tel: 0091-11-26735119, 0091-11-26742750; Fax: 0091-11-2674165;

Email: asis_datta@rediffmail.com

Gene organization of *FvCaMLP*

Complete Fv*CaMLP* gene was amplified by PCR using genomic DNA of *F. velutipes* as template and sequenced. Primers used for amplification were designed using the sequence information derived from cDNA clone of Fv*CaMLP* obtained during yeast one hybrid as well as 5' untranslated sequence obtained by genome walking (supplementary methods). Comparison of the genomic DNA and cDNA sequence of Fv*CaMLP* showed that 681bp of Fv*CaMLP* gene was interrupted by five introns. Splice donor and acceptor sites in introns were typical of eukaryotes with GT and AG at 5' and 3' end, respectively (Fig. S2a). The first intron in Fv*CaMLP* gene is a phase zero intron that lies immediately after ATG[1]. Presence of five introns and positioning of first intron immediately after initiation codon is a feature conserved in other reported fungal calmodulins like that of *H. capsulatum*, *P. brasiliensis, A. nidulans* etc.[2]. The second intron lies close to first, separated by 13base pairs. Second, third and fourth introns are phase one introns, whereas fifth one is phase two intron. Like most fungal calmodulins, Fv*CaMLP* was determined to be a single copy gene (Fig. S2b) as confirmed by Southern blot analysis (supplementary methods) using suitable restriction enzymes.

Table S1. Strains and plasmids used in this study

Strains and plasmids	Genotype/comments	Source/reference
***E. coli* strains**		
DH5α	F'/end A1 hsd R17 ($r_K^- m_k^+$) glnV44 thi-4 - recA1 gyrA (Nal^n) elA1 Δ(lac1ZYA-argF) U169 deoR (ϕ80dlac Δ(lacZ) M15)	Laboratory strain
BL21 (DE3)	*E. coli* B F^- dcm ompT hsdS ($rB^- mB^-$) gal--λ (DE3)	Laboratory strain
F. velutipes strain		
ATCC13547		ATCC
S. pombe strain		
BJ7468	ura4-D18, leu1-32, and ade6-M216	Laboratory strain
**Plasmids**		
pFv*CaMLP*	Fv*CaMLP* cDNA cloned in pGEM-TEasy	this study
pGST-*FvCaMLP*	Fv*CaMLP* cDNA cloned in pGEX4T-2	this study
pTN54	GFP in pREP41, LEU2 based vector with thiamine repressible nmt41 promoter	Nakamura *et al*[3]
pGFP-Fv*CaMLP*	Fv*CaMLP* cDNA cloned in pTN54	this study
pGFP-Fv*CaMLP*ΔNES	Fv*CaMLP*ΔNES cDNA cloned in pTN54	this study
pHis-OxPro	500bp of 5'upstream region of *OXDC* gene cloned in pHis2.1	this study

Table S2. Probable interacting partners of FvCamlp

**Interacting Partner**	**Template Structure**	**Interacting Z-value**	**Interacting Score**	**Description**	**Organism**
P19524	1n2dAC	1.77	301.89	Myosin-2	*S. cerevisiae*
Q9USI6	1oe9AB	6.04	248.13	Myosin type-2 heavy chain 1	*S. pombe*
P32492	1oe9AB	5.66	237.01	Myosin-4.	*S. cerevisiae*
O14157	2bl0AC	5.62	361.94	Myosin type-2 heavy chain 2	*S. pombe*
P36006	1oe9AB	5.52	232.60	Myosin-3	*S. cerevisiae*
Q9Y7Z8	1oe9AB	5.51	232.55	Myosin-1.	*S. pombe*
Q04439	1oe9AB	5.27	225.30	Myosin-5.	*S. cerevisiae*
O74805	1oe9AB	4.72	208.91	Myosin-51	*S. pombe*
P08964	1oe9AB	4.37	198.48	Myosin-1	*S. cerevisiae*
P14747	1auiAB	3.81	170.43	Serine/threonine-protein phosphatise (calcineurin) 2B catalytic subunit A2	*S. cerevisiae*
P23287	1auiAB	3.62	165.92	Serine/threonine-protein phosphatise (calcineurin) 2B catalytic subunit A2	*S. cerevisiae*
Q12705	1auiAB	3.51	163.34	Serine/threonine-protein phosphatase (calcineurin)2B catalytic subunit A2	*S. pombe*
P48457	1auiAB	3.34	159.05	Serine/threonine-protein phosphatise (calcineurin) 2B catalytic subunit A2	*E. nidulans*
Q05681	1auiAB	3.25	156.83	Serine/threonine-protein phosphatise (calcineurin) 2B catalytic subunit A2	*N. crassa*

Table S3. List of synthetic siRNA targeting Fv*CaMLP*

	**Sense**	**antisense**
1	GAGCUGAGAUACGUUCUUAtt	UAAGAACGUAUCUCAGCUCtt
2	GUUGCCACCAUCGUUUCAUtt	AUGAAACGAUGGUGGCAACtt
3	CGACAACGCAGAAUACAAAtt	UUUGUAUUCUGCGUUGUCGtt
4	CAGACCAGAUGGCUUCAAUtt	AUUGAAGCCAUCUGGUCUGtt
5	GAACACCAGAGGAAUUCAUtt	AUGAAUUCCUCUGGUGUUCtt
6	GGCAACGUCAAUUACGAGUtt	ACUCGUAAUUGACGUUGCCtt
7	CACCAAAGGAUGUGGACUAtt	UAGUCCACAUCCUUUGGUGtt

Table S4. List of proteins interacting to FvCamlp as identified by MALDI analysis

Band no.	Accession	Mowse score	Mass	Sequence coverage	Description	Peptides matched (shown in red)
FvCamlp	**gi|299739032**	74	15496	50%	myosin regulatory light chain cdc4 [Coprinopsis cinerea okayama]	**MSDNAEYKEAFALFDKR**GTG AVPREVLGDLLRALGQNPTQ<br>AEVAEIVASAPR**DVDYKTFLTILNRPDGFKPAGTPEEFIR**GF<br>QVFDKEGNGFIGAGELR**YVLTQLGEK**MTDEEVDELLK**GVQIG**<br>**ADGNVNYESFVR**TILSQ
Interacting protein 1<br><br>(Band 1)	**gi|121710522**	46	41784	26%	protein kinase, putative [Aspergillus clavatus NRRL 1]	MGDAFNAEIHPVYLGQVLHHRYRILQRLGSG**RYSAVYLARDQ**<br>**KESSYKA**IKILKPDCYDGKHDLFELEILRHLSRANPDHPGYQ<br>HITILLDDFIHIGQSGNRHVCLVMEPMAEDM**KSFSFFFDGAK**<br>**IPNRIMKQITKQ**LLSALEYAHASGIIHTDI**KQDNIMVKIRNP**<br>**STIDRY**LEDSLSNSTANLGEYNFDDTSELVHTDVVLCDWGSA<br>SWVRKHLTEMIQPKLLRAPEVIIGAPWGKEVDIWNLGALLPE<br>LLDTVQMFSGKANVTGGIYHIKHHIEEIDALFGPFPSEMLAN<br>GNP**KLVEHIFDKNSNIRDRT**KRPPAMLERWIQSLDGAEK**TNF**<br>**LSLIRSMLIINPKERK**SAMSLQCASWLTV
Interacting protein 2<br><br>(Band 2)	**gi|134109773**	55	30460	27%	hypothetical protein CNBC4910 [Cryptococcus neoformans var. neoformans	**MFTNLSPEDK**DAFFSLLDEYFASRPHNLPSPPAIDSSSHPAR<br>SNAALSPPPSAGYASPSYSSAR**HPMPPPERTEQPDSAQR**FIS<br>SSIKYGTAGTK**SSMNAVSKNK**DAMDLLGKVGMSSMVGR**ANER**<br>**MNKPAEAGVKEEAGRKAAPPPIAAKK**GGVSGLVSSR**MSAFTS**<br>**MWRDPQK**SKPPAVEQTISPTLSYSNTALPPPIRRDGSGSYQS<br>SSPNPASSAAAEVGPELANGDEGQAQALYDYTGNDKGDLS<br>VQANQVVNIIEKTSSDWWTCEDGNGQRGLVPATYLQAI
Interacting protein 3<br><br>(Band 3)	**gi|156030438**	73	25640	49%	hypothetical protein SS1G_14522 [Sclerotinia sclerotiorum 1980]	**MSIVPAGIEMR**RNAMYNLTDVSTPGDDVYVPQQTKCTWEVAF<br>RYISR**MYRPLAMTWVAQR**QTTEEEDLVYCLLGLCEVSMPPIY<br>GEGKEVALK**RLEMTVKGFSTNESEPKDLKDNTASFIVPFGRN**<br>**LNFIGRGTQLTEVEAKLFTGGR**MTK**VAITGLGGIGKTQLLLE**<br>**LVYRIR**DRYKDCLVIWIPATNTESLHQAYREVARQLKIPGSD<br>EDK**ADGKETW CK**II

Figure legends

Figure S1 | (a) Morphological response of *F. velutipes* on PDA with or without oxalic acid (5mM). (b) Light microscopy image of fungal mycelia grown on PDA with or without oxalic acid (5mM). (c) Growth on PDA plate (-oxalic acid) with and without siRNA against Fv*CaMLP*.

Figure S2 | Fv*CaMLP* is a single copy gene with five introns. (a) Nucleotide and the deduced amino acid sequence of the Fv*CaMLP* gene**.** An open reading frame of 426 bp and the predicted 141 amino acids residues are shown. The exons are indicated by upper case letters and the introns and non coding regions are indicated by lower case letters. Initiation and termination codons ATG and TGA, respectively, are in bold letters. The intron junctions GT/AG are highlighted in light grey boxes. (b) Southern blot analysis to determine copy number of Fv*CaMLP*. 5.0μg of genomic DNA from *F. velutipes* was used for restriction digestion with 1, *SacI*., 2, *SacII*., 3, *XbaI*., 5, *BanI*., and 4, uncut genomic DNA. [α-P^{32}]CTP labeled complete Fv*CaMLP* gene was used as probe. Positions of different size fragments of 1kb ladder are shown at left.

Figure S3| Potential phosphorylation sites in FvCamlp. *In silico* prediction of serine/ threonine phosphorylation sites in FvCamlp by Net Phos 2.0 server[4].

Figure S4| GST pull down assay to detect the interacting partners of FvCamlp. SDS PAGE analysis showed three proteins (depicted as 1, 2, 3) interacting to FvCamlp-GST fusion protein. No interacting protein could be detected in GST negative control. M, molecular weight marker

Supplementary methods

Southern blot analysis. Genomic DNA was isolated using cetyltrimethylammonium bromide (CTAB) method. 5μg of digested DNA was separated on 0.8% agarose gel and Southern blot analysis was performed as described in [5]. High stringency post-hybridization washes were performed at 65 °C with 2 x SSC, 1% w/ v SDS and with 0.2 x SSC, 1% w/ v SDS.

Genome walking. For cloning of Fv*CaMLP* promoter, Genome Walker Universal kit (Clontech) was used as per the manufacturer's instructions. Four genomic DNA libraries were constructed using high quality genomic DNA isolated from *F. velutipes* and digested with four different blunt end restriction enzymes ECoRV, DraI, StuI and PvuII followed by adapter ligation of genomic DNA fragments. After the library construction, primary PCR was carried out using the outer adapter primer (AP1: 5'-GTAATACGACTCACTATAGGGC-3') provided with the kit and gene specific primer1 (5′-CGGATTCTGTCCGAGGGCGCGAAGCAG-3′).The primary PCR mix was then diluted and used as template for secondary or nested PCR using nested adapter primer (AP2: 5'–ACTATAGGGCACGCGTGGT–3') and nested gene specific primer 2 (5′GCGCCTGTACCGCGTTTATCGAAGAGCG3′). The amplicons, thus obtained were gel eluted and ligated in pGEM-TEasy vector (Promega) followed by sequencing.

GST pull down assay and MALDI (Matrix- assisted laser desorption ionization) analysis of interacting proteins

GST pull down assay was performed as described previously [6].Total protein extract from *F. velutipes* was applied to glutathione sepharose matrix on which either purified FvCamlp-GST fusion protein or GST negative control has been immobilized and incubated for 3.0 hours.

After washing the matrix 3-5 times with 1X PBS, interacting proteins were eluted with 20mM reduced glutathione in 50mM Tris-HCl (pH 8.0) and resolved on 10% SDS- PAGE gel. Gel was silver stained and desired bands were excised. Gel pieces were subjected to in-gel trypsin digestion as described earlier[7]. Interacting protein were identified by MALDI using ABSCIEX 4800 MALDI TOF/ TOF analyzer. Data was analyzed using GPS explorer TM software version 3.6.

Supplementary References

1. Fedorov, A. et al. Analysis of non uniformity in intron phase distribution. *Nucleic Acids Res.* **20**, 2553-2557 (1992).
2. Carvalho, M.J. et al. Functional and genetic characterization of calmodulin from the dimorphic and pathogenic fungus *Paracoccidioides brasiliensis*. *Fungal Genet Biol.* **39**, 204-210 (2003).
3. Nakamura, T., Nakamura-Kubo, M., Hirata, A., and Shimoda, C. The *Schizosaccharomyces pombe* spo3^{+} gene is required for assembly of the forespore membrane and genetically interacts with psy1^{+}-encoding syntaxin-like protein. *Mol Biol Cell.* **12,** 3955-3972(2001).
4. Blom, N., Gammeltoft, S. & Brunak, S. Sequence- and structure-based prediction of eukaryotic protein phosphorylation sites. *J Mol Biol*. **294**, 1351-1362 (1999).
5. Southern, E. M .Detection of specific sequences among DNA fragments separated by electrophoresis. *J Mol Biol* **98**, 503- 517(1975).
6. Orlinick, J.R. & Chao, M.V. Interactions of the cellular polypeptides with the cytoplasmic domain of the mouse Fas antigen. *J Biol Chem.* **271,** 8627–8632 (1996).

7. Shevchenko, A. et al. In-gel digestion for mass spectrometric characterization of proteins and proteomes. *Nat Prot.***1**, 2856-2860 (2006).

Supplementary Figure 1

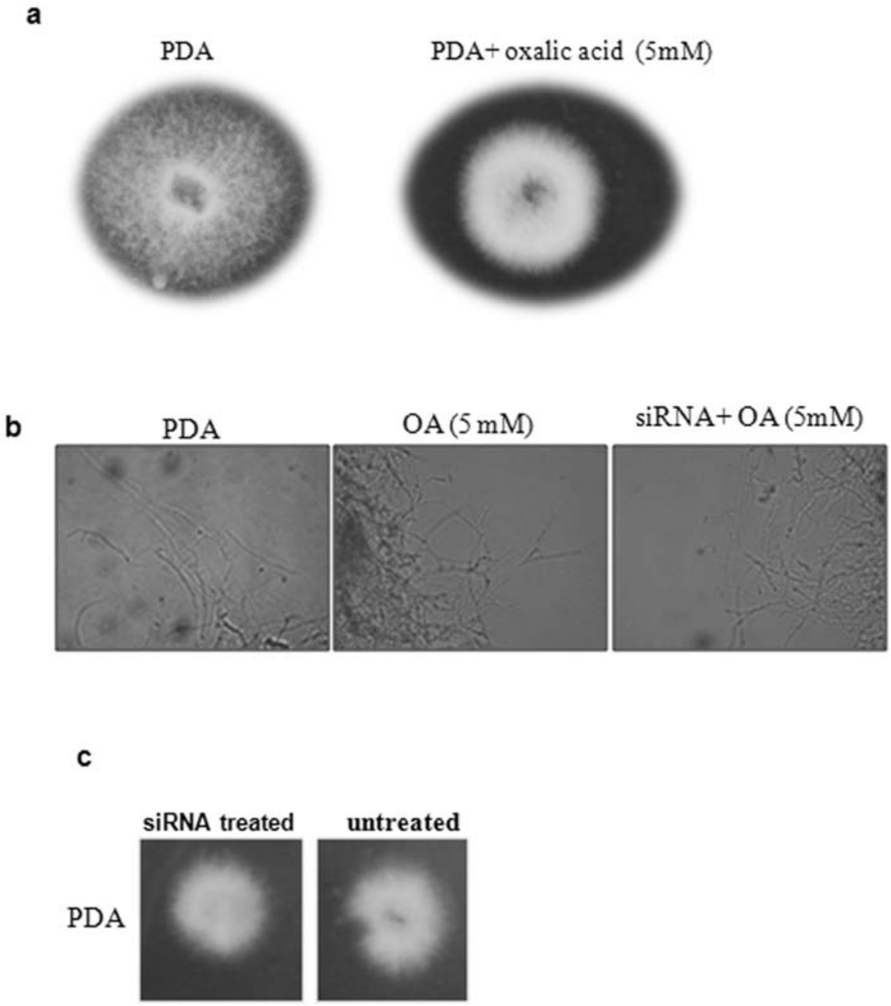

Supplementary Figure 2

a

```
-978 atcctgcccattgataccgaacaataggtggtggacaatctccatagtgt

-928 tttcaggccacgtcgctgagtcgtcatccgaggatggtagcctggacggg

-878 gccagcgacttgggaagaggtggcggaatacgcgaaagtgggaggaggag

-828 ggcggtggttgagacgactggagaggacgccgaaaagttggcgataaaag

-778 aggcagatgctgtggttaaaagatttgggggtgccaacgcatcagcgaga

-728 gacgcctagaatggaaaacgggagtatgcaacctggcaatatgatgatac

-678 acttaccacaccagctgggaccttgcctgtagaaaggtagcgaatgggag

-628 ctgcatagaaaggaataggctccaatgatatataggacggcacagggtac

-578 acgtcaagcagagtgacactcgtcggttccaattcttgcgccaccctttt

-528 tcacgtatggatctatagccgagatgggcaggcgcgtggtcgcttcagac

-478 acaaggattgtagattgcgaccatgctggagtgaacaaaaggcccaccta

-428 gatattcgttggctcggtcatgccatgaatgaatgtgaacgcactgcgac

-378 agtgttcacgtagacgcccgcggtctgttctcccagatctgcgccgcggg

-328 cccaatatttccctgcatcacctgatgcgacgaccagagggcgtcctttg

-278 gggatgtcgccgatgagtttgatttcccaatctacgactggctcggattt

-228 ggatggagtttcgagaggattgtattcgtcctcttcgtcagattcgacag

-178 cgtagcgcggtggcacagtatcttcaaggggatcgacatcctagagggca

-128 cagtgagcatggattgcagggagacacagtgcgacctgccatgatgaatg

 -78 ttggaagcaaacgttacgcgtctcggcaaagcccgacgaatctcaacctc

 -28 caacaaccatctctttcaacactttgca

   1 ATGgtcagctctatccgtctccagtttgacgtcttactgaactaatacac
     M
  51 agAGCGACAACGCAGgttaacatccatcctacaaggctttaagattactg
       S  D  N  A  E
 101 gatctaacactaagcaacagAATACAAAGAGGCCTTTGCGCTCTTCGATA
                           Y  K  E  A  F  A  L  F  D  K
 151 AACGCGGTACAGGCGCTGTTCCCCGCGAAACGCTCGGGGATCTGCTTCGC
       R  G  T  G  A  V  P  R  E  T  L  G  D  L  L  R
 201 GCCCTCGGACAGAATCCGACACAGGCAGACGTTGCCACCATCGTTTCATC
     A  L  G  Q  N  P  T  Q  A  D  V  A  T  I  V  S  S
 251 GGCACCAAAGGATGgtcagcattccctttcccctctccatctattccatc
      A  P  K  D  V
 301 tcacgcgtgcacaccaagTGGACTACAAAACCTTCCTTACGATCCTAAAC
                         D  Y  K  T  F  L  T  I  L  N
 351 AGACCAGATGGCTTCAATCCTGCCGGAACACCAGgtgcgttgtcattgaa
     R  P  D  G  F  N  P  A  G  T  P  E
 401 ctatgcacaaaaccatctcataccagtctagAGGAATTCATCCGCGGTTT
```

```
                                      E  F  I  R  G  F
451 CCAAGTGTTCGACAAGGAGCACAATGGATTCATCGGTGCTGGAGAGCTGA
     Q  V  F  D  K  E  H  N  G  F  I  G  A  G  E  L  R
501 GATACGTTCTTACACAACTGGGCGAGAAGATGTCGGACGAGGAGGTTGAT
      Y  V  L  T  Q  L  G  E  K  M  S  D  E  E  V  D
551 GAGCTCTTGAAGGGTGTCCAAATTGGCGCgtacgtgcttttgcattgtcc
    E  L  L  K  G  V  Q  I  G  A
601 ttacgctgaaatgttgactgtttgaacagCGACGGCAACGTCAATTACGA
                                   D  G  N  V  N  Y  E
651 GTCCTTTGTACGCACTATCCTCAGCCAATGA
     S   F V  R   T  I  L S   Q
```

Supplementary Figure 2

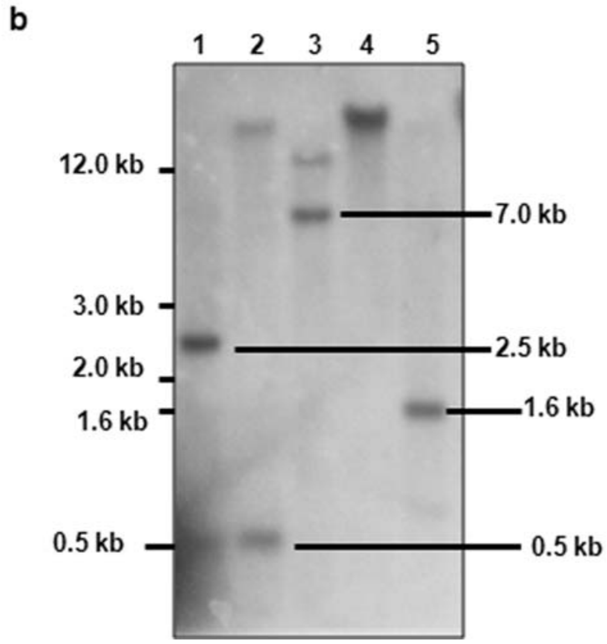

Supplementary Figure 3

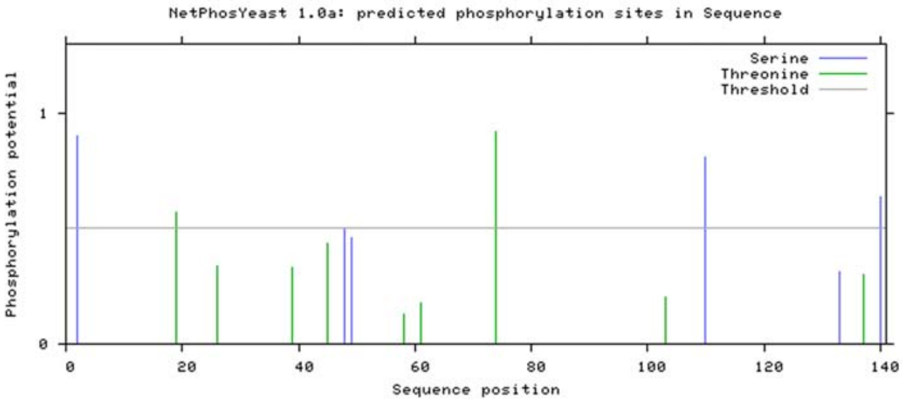

Supplementary Figure 4

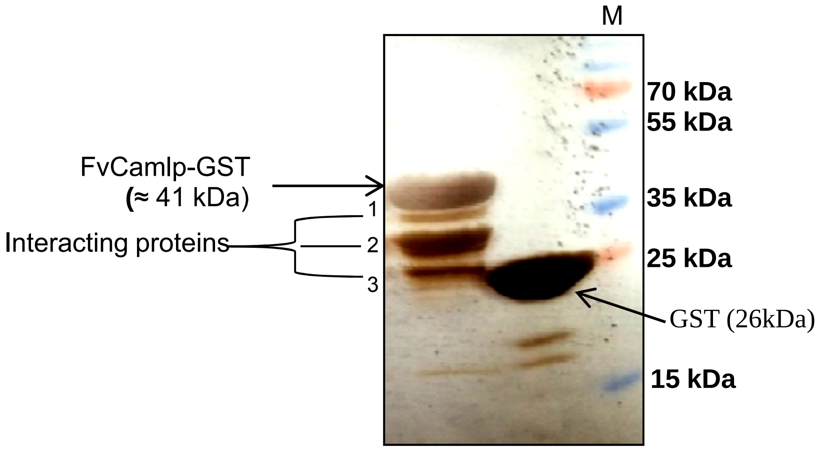

Unprocessed images

Figure 1 d

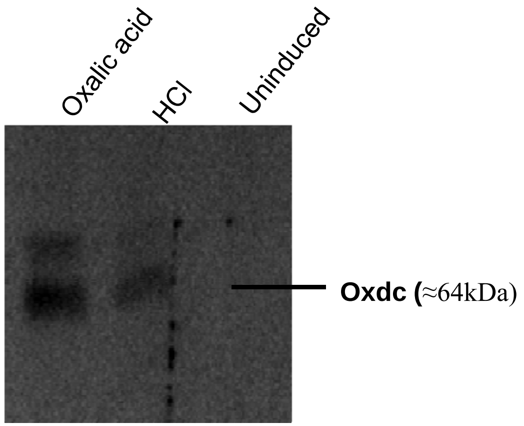

Figure 3b

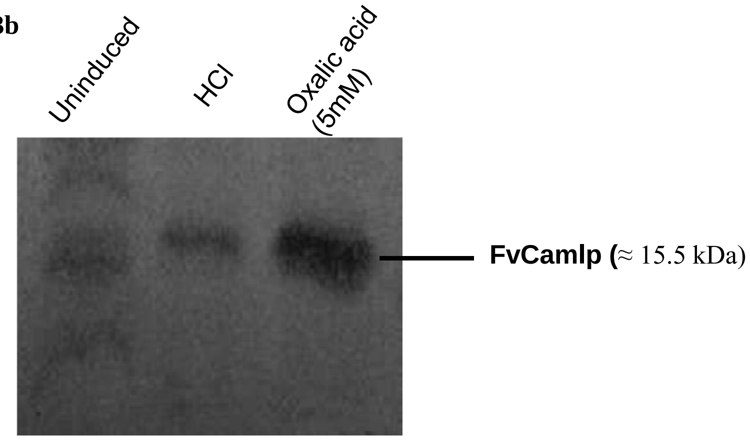

Figure 4a

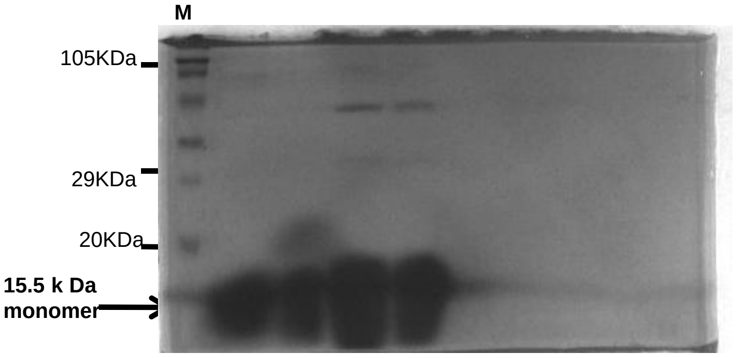

Figure 4 g

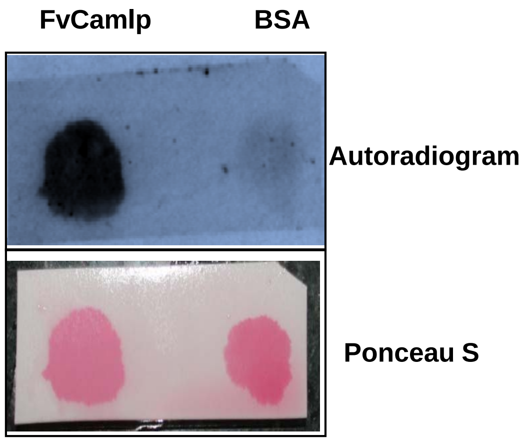

Figure 4 e

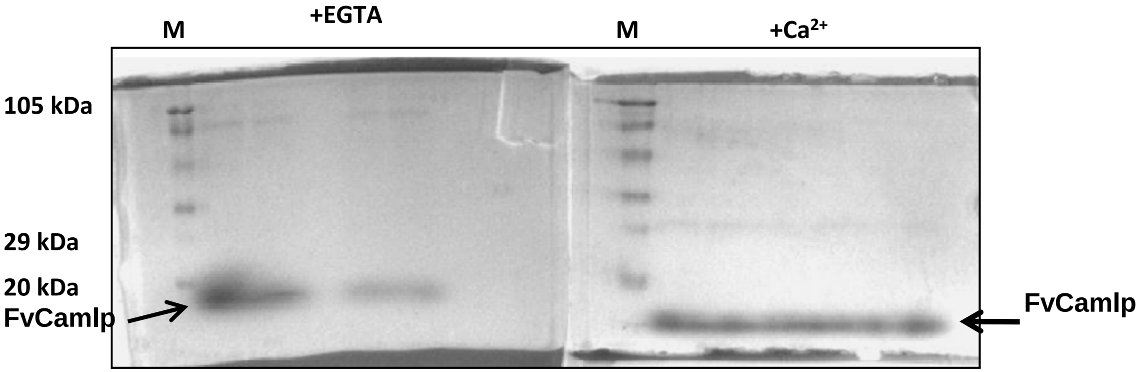

Figure 5 b

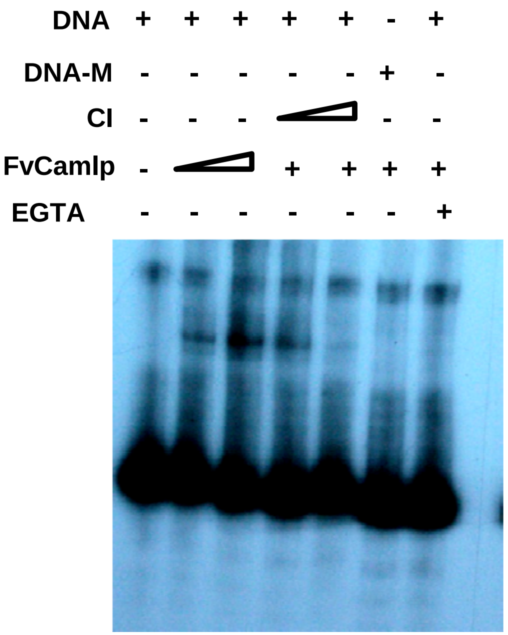

Figure 5 d

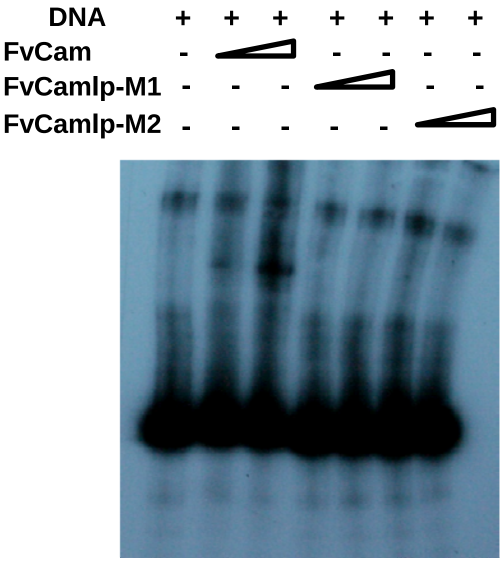

Supplement: Supplementary Information [file srep14578-s1.pdf]
